# Supplementary figures and images for: A titin missense variant drives atrial electrical remodeling and is associated with atrial fibrillation
Source: eLife. 2026 Jan 22;14:RP104719. doi: 10.7554/eLife.104719 (PMC12826672; doi:10.7554/eLife.104719)

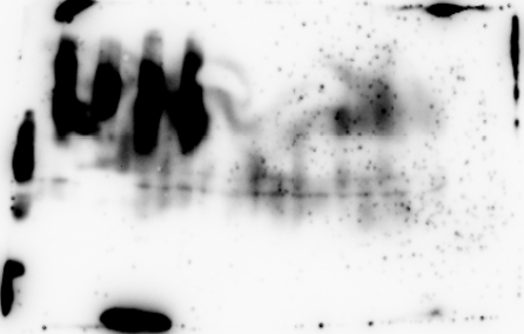

Supplement: Figure 5—source data 1. [file elife-104719-fig5-data1.zip › FHL2 raw image.tif]

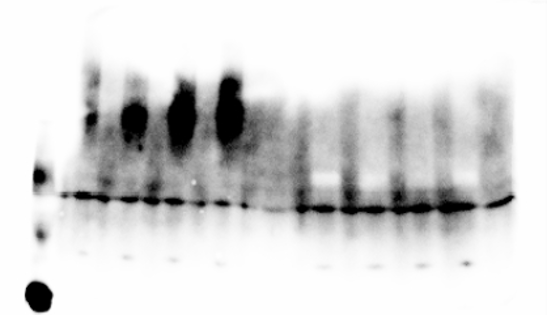

Supplement: Figure 5—source data 1. [file elife-104719-fig5-data1.zip › MinK raw image.tif]

# FHL2-MinK CO-IP

MinK

27kD

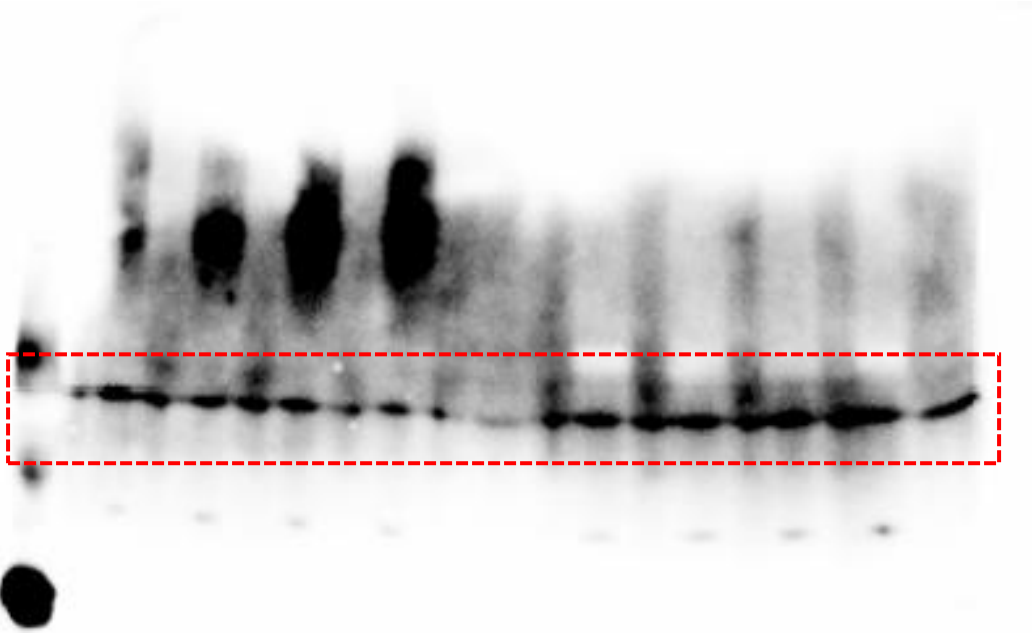

FHL2

32 kD

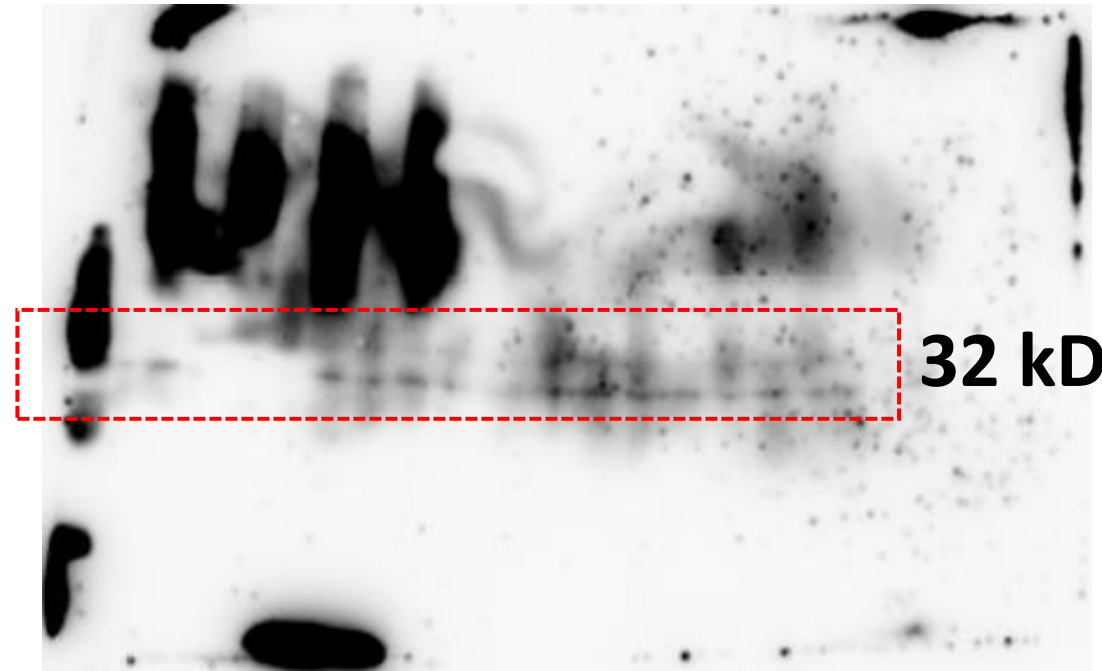

Supplement: Figure 5—source data 2. [file elife-104719-fig5-data2.zip › Western blot CO-IP raw images.pdf]
