# Supplementary material for: A titin missense variant drives atrial electrical remodeling and is associated with atrial fibrillation
Source: eLife. 2026 Jan 22;14:RP104719. doi: 10.7554/eLife.104719 (PMC12826672; doi:10.7554/eLife.104719)
Supplement: Supplementary file 1. — Age represents patient’s age at AF diagnosis in years. M=male, F=female, HL = Hispanic/Latinx, NHB = non-Hispanic Black. Variants with a blank value in the dbSNP or gnomAD columns represent variants not present in those respective databases. [file elife-104719-supp1.docx]

| **Subject ID** | **Age** | **Sex** | **Race-**  **Ethnicity** | **Nucleotide** | **Amino Acid**  **Change** | **dbSNP** | **gnomAD**  **Allele Frequency** | **Exon** | **Band** | **Percent Spliced**  **In (PSI)** | **REVEL Score** |
| --- | --- | --- | --- | --- | --- | --- | --- | --- | --- | --- | --- |
| 39 | 56 | F | NHB | c.70250T>C | p.Ile23417Thr | rs201836227 | 0.000221 | 326 | A-band | 100 | 0.66297 |
| 51 | 50 | M | HL | c.52022G>A | p.Arg17341Gln | rs370390570 | 0.000116 | 273 | A-band | 100 | 0.60691 |
| 51 | 50 | M | HL | c.62519G>A | p.Gly20840Asp | rs1326564200 | 0.000012 | 304 | A-band | 100 | 0.883 |
| 54 | 57 | M | NHB | c.59248G>A | p.Gly19750Ser | rs200732032 | 0.000109 | 300 | A-band | 100 | 0.75279 |
| 54 | 57 | M | NHB | c.58363G>A | p.Gly19455Ser | rs191927501 | 0.000157 | 297 | A-band | 100 | 0.80842 |
| 60 | 66 | M | HL | c.57727G>C | p.Ala19243Pro | rs1313667626 | 0.000004 | 295 | A-band | 100 | 0.81369 |
| 65 | 43 | F | NHB | c.101665G>A | p.Val33889Ile | rs34924609 | 0.003099 | 358 | A-band | 100 | 0.17295 |
| 65 | 43 | F | NHB | c.6959G>A | p.Arg2320His | rs374615369 | 0.000076 | 30 | I-band | 100 | 0.82403 |
| 71 | 58 | M | NHB | c.42145G>T | p.Val14049Leu | rs1206523368 | |  | I-band | 100 | 0.73517 |
| 72 | 62 | M | NHB | c.67808C>T | p.Ala22603Val | rs199583938 | 0.000036 | 320 | A-band | 100 | 0.42028 |
| 72 | 62 | M | NHB | c.101557A>G | p.Lys33853Glu | rs727505163 | 0.000004 | 358 | A-band | 100 | 0.75074 |
| 80 | 76 | F | NHB | c.93266G>A | p.Arg31089His | rs367993101 | 0.000028 | 339 | A-band | 100 | 0.87283 |
| 80 | 76 | F | NHB | c.970C>T | p.Pro324Ser | rs72647845 | 0.000598 | 7 | Z-disk | 100 | 0.70603 |
| 87 | 56 | M | HL | c.89426G>A | p.Arg29809Gln | rs72648238 | 0.000632 | 334 | A-band | 100 | 0.45592 |
| 87 | 56 | M | HL | c.99433C>T | p.Arg33145Trp | rs1338284042 | 0.000004 | 355 | A-band | 100 | 0.61955 |
| 96 | 77 | M | NHB | c.74870A>G | p.Lys24957Arg | rs760043791 | 0.000004 | 326 | A-band | 100 | 0.43483 |
| 96 | 77 | M | NHB | c.70817T>C | p.Met23606Thr | rs371030086 | 0.000040 | 326 | A-band | 100 | 0.624 |
| 116 | 83 | F | NHB | c.101665G>A | p.Val33889Ile | rs34924609 | 0.003099 | 358 | A-band | 100 | 0.17295 |
| 129 | 36 | F | NHB | c.96605T>C | p.Val32202Ala | rs763365622 | 0.000004 | 347 | A-band | 100 | 0.54781 |
| 148 | 35 | F | NHB | c.70543T>G | p.Tyr23515Asp | |  |  | A-band | 100 | 0.67962 |
| 150 | 65 | M | NHB | c.4671G>A | p.Met1557Ile | rs139192633 | 0.000272 | 27 | near Z-disk | 100 | 0.26882 |
| 160 | 69 | F | NHB | c.91937A>G | p.Asn30646Ser | rs72648245 | 0.000568 | 338 | A-band | 100 | 0.71558 |
| 160 | 69 | F | NHB | c.46693G>T | p.Ala15565Ser | rs145520397 | 0.000445 | 250 | I-band | 100 | 0.51417 |
| 164 | 68 | F | NHB | c.100396C>T | p.Arg33466Cys | rs371908649 | 0.000145 | 357 | A-band | 100 | 0.8849 |
| 164 | 68 | F | NHB | c.56693G>A | p.Arg18898His | rs572453785 | 0.000051 | 291 | A-band | 100 | 0.29647 |
| 167 | 46 | M | NHB | c.82061T>G | p.Val27354Gly | rs368023868 | 0.000036 | 326 | A-band | 100 | 0.77847 |
| 167 | 46 | M | NHB | c.76987G>A | p.Asp25663Asn | rs143186270 | 0.000105 | 326 | A-band | 100 | 0.49396 |
| 173 | 84 | F | NHB | c.8938G>A | p.Ala2980Thr | rs72647885 | 0.000371 | 38 | I-band | 100 | 0.75822 |
| 189 | 70 | F | NHB | c.9077A>T | p.Asn3026Ile | rs11900987 | 0.000454 | 38 | I-band | 100 | 0.56024 |
| 198 | 85 | F | HL | c.7180G>C | p.Glu2394Gln | rs537269762 | 0.000004 | 31 | I-band | 100 | 0.58254 |
| 205 | 46 | M | HL | c.57586C>G | p.Leu19196Val | rs397517630 | 0.000171 | 295 | A-band | 100 | 0.50185 |
| 235 | 62 | F | NHB | c.44072C>T | p.Thr14691Ile | rs1048028645 | |  | I-band | 100 | 0.73445 |
| 235 | 62 | F | NHB | c.63245C>A | p.Thr21082Asn | |  |  | A-band | 100 | 0.74377 |
| 240 | 66 | F | NHB | c.101936C>G | p.Pro33979Arg | rs200238877 | 0.000213 | 358 | A-band | 100 | 0.73517 |
| 240 | 66 | F | NHB | c.57683G>A | p.Arg19228His | rs114711705 | 0.000488 | 295 | A-band | 100 | 0.30369 |
| 240 | 66 | F | NHB | c.85691A>T | p.Lys28564Ile | rs199859344 | 0.000443 | 326 | A-band | 100 | 0.69443 |
| 243 | 57 | M | NHB | c.44525C>T | p.Thr14842Ile | rs370782364 | 0.000012 | 241 | I-band | 100 | 0.75347 |
| 243 | 57 | M | NHB | c.81502C>T | p.Arg27168Cys | rs377616334 | 0.000028 | 326 | A-band | 100 | 0.63269 |
| 358 | 68 | F | NHB | c.88340C>G | p.Thr29447Arg | rs140201636 | 0.000198 | 331 | A-band | 100 | 0.58883 |
| 358 | 68 | F | NHB | c.87137T>G | p.Met29046Arg | rs143975327 | 0.000192 | 328 | A-band | 100 | 0.81427 |
| 358 | 68 | F | NHB | c.73316C>T | p.Thr24439Ile | rs750110781 | 0.000008 | 326 | A-band | 100 | 0.32913 |
| 395 | 60 | F | NHB | c.98267C>T | p.Thr32756Ile | rs199805060 | 0.000330 | 352 | A-band | 100 | 0.58758 |
| 396 | 63 | M | HL | c.91573A>G | p.Ile30525Val | rs72648244 | 0.006222 | 337 | A-band | 100 | 0.18881 |
| 396 | 63 | M | HL | c.72931A>G | p.Thr24311Ala | rs56201325 | 0.003985 | 326 | A-band | 100 | 0.1533 |
| 400 | 62 | F | NHB | c.8938G>A | p.Ala2980Thr | rs72647885 | 0.000371 | 38 | I-band | 100 | 0.75822 |
| 412 | 66 | M | NHB | c.79612A>G | p.Thr26538Ala | rs150682764 | 0.000322 | 326 | A-band | 100 | 0.70029 |
| 413 | 67 | M | HL | c.14911T>G | p.Cys4971Gly | rs537312655 | 0.000439 | 50 | I-band | 100 | 0.3358 |
| 426 | 57 | M | NHB | c.43622C>T | p.Ser14541Leu | rs768180052 | 0.000008 | 236 | I-band | 100 | 0.60219 |
| 457 | 42 | F | NHB | c.69883G>A | p.Ala23295Thr | rs746519147 | 0.000032 | 326 | A-band | 100 | 0.21907 |
| 459 | 78 | M | NHB | c.52927C>T | p.Arg17643Trp | rs375944265 | 0.000060 | 276 | A-band | 100 | 0.75279 |
| 459 | 78 | M | NHB | c.103363C>T | p.Arg34455Cys | rs72629785 | 0.000716 | 358 | A-band | 100 | 0.75687 |
| 490 | 76 | M | NHB | c.81539T>C | p.Ile27180Thr | rs182126530 | 0.000669 | 326 | A-band | 100 | 0.64318 |
| 490 | 76 | M | NHB | c.44077C>T | p.Arg14693Cys | rs200445568 | 0.000169 | 238 | I-band | 100 | 0.68844 |
| 493 | 63 | M | NHB | c.103363C>T | p.Arg34455Cys | rs72629785 | 0.000716 | 358 | A-band | 100 | 0.75687 |
| 498 | 77 | M | NHB | c.61481T>C | p.Ile20494Thr | rs374845737 | 0.000022 | 304 | A-band | 100 | 0.7293 |
| 506 | 52 | F | NHB | c.54348A>T | p.Glu18116Asp | rs773746281 | 0.000016 | 281 | A-band | 100 | 0.48268 |
| 508 | 72 | M | NHB | c.103906C>T | p.Arg34636Cys | rs768575577 | 0.000028 | 358 | A-band | 100 | 0.59861 |
| 508 | 72 | M | NHB | c.97892A>T | p.Lys32631Ile | rs944963846 | 0.000004 | 351 | A-band | 100 | 0.56959 |
| 513 | 51 | F | NHB | c.2765G>A | p.Arg922His | rs56046320 | 0.000703 | 16 | near Z-disk | 99 | 0.27141 |
| 513 | 51 | F | NHB | c.60104G>A | p.Cys20035Tyr | rs774488793 | |  | A-band | 100 | 0.71869 |
| 513 | 51 | F | NHB | c.55079C>T | p.Pro18360Leu | rs192788942 | 0.000117 | 283 | A-band | 100 | 0.76881 |
| 521 | 94 | M | HL | c.95414T>G | p.Phe31805Cys | |  |  | A-band | 100 | 0.73005 |
| 521 | 94 | M | HL | c.74504A>G | p.Tyr24835Cys | rs201724962 | 0.000069 | 326 | A-band | 100 | 0.80901 |
| 540 | 57 | M | NHB | c.78896T>A | p.Val26299Asp | rs73036377 | 0.000131 | 326 | A-band | 100 | 0.84613 |
| 542 | 46 | M | NHB | c.101245G>A | p.Val33749Met | rs201554140 | 0.000538 | 358 | A-band | 100 | 0.72631 |
| 542 | 46 | M | NHB | c.4199G>C | p.Ser1400Thr | rs138506461 | 0.000518 | 24 | near Z-disk | 100 | 0.12618 |
| 542 | 46 | M | NHB | c.105127C>T | p.Arg35043Cys | rs200378865 | 0.000462 | 358 | A-band | 100 | 0.79765 |
| 544 | 51 | M | NHB | c.2599A>G | p.Ser867Gly | rs148631577 | 0.000084 | 16 | near Z-disk | 99 | 0.27398 |
| 544 | 51 | M | NHB | c.970C>T | p.Pro324Ser | rs72647845 | 0.000598 | 7 | Z-disk | 100 | 0.70603 |
| 556 | 59 | F | NHB | c.69130C>T | p.Pro23044Ser | rs55980498 | 0.003619 | 324 | A-band | 100 | 0.83802 |
| 586 | 55 | M | HL | c.95557C>A | p.Arg31853Ser | |  |  | A-band | 100 | 0.47777 |
| 586 | 55 | M | HL | c.67989A>T | p.Leu22663Phe | rs1485610846 | 0.000004 | 320 | A-band | 100 | 0.71401 |
| 632 | 47 | M | HL | c.88394C>T | p.Ser29465Phe | rs146181116 | 0.002928 | 331 | A-band | 100 | 0.65325 |
| 632 | 47 | M | HL | c.89314G>A | p.Glu29772Lys | rs200503016 | 0.000245 | 334 | A-band | 100 | 0.5787 |
| 647 | 64 | M | NHB | c.47737C>T | p.Leu15913Phe | rs138576504 | 0.000399 | 254 | A-band | 100 | 0.60456 |
| 647 | 64 | M | NHB | c.98893G>A | p.Asp32965Asn | rs186405108 | 0.000044 | 353 | A-band | 100 | 0.68757 |
| 650 | 72 | M | HL | c.93392T>G | p.Val31131Gly | rs1176407616 | 0.000012 | 339 | A-band | 100 | 0.47942 |
| 659 | 44 | F | NHB | c.97760G>A | p.Arg32587His | rs55704830 | 0.001734 | 350 | A-band | 100 | 0.50341 |
| 667 | 57 | F | NHB | c.95876T>A | p.Val31959Glu | rs761732372 | 0.000008 | 345 | A-band | 100 | 0.70924 |
| 693 | 95 | F | HL | c.83870G>C | p.Arg27957Thr | rs148067743 | 0.000145 | 326 | A-band | 100 | 0.23913 |
| 698 | 53 | F | NHB | c.61322A>G | p.Asn20441Ser | rs147580753 | 0.000260 | 304 | A-band | 100 | 0.34673 |
| 698 | 53 | F | NHB | c.8938G>A | p.Ala2980Thr | rs72647885 | 0.000371 | 38 | I-band | 100 | 0.75822 |
| 704 | 70 | F | HL | c.102030T>G | p.Ser34010Arg | rs1296387134 | 0.000024 | 358 | A-band | 100 | 0.69273 |
| 738 | 62 | F | HL | c.102427A>T | p.Met34143Leu | rs371226574 | 0.000004 | 358 | A-band | 100 | 0.46781 |
| 738 | 62 | F | HL | c.96928A>C | p.Thr32310Pro | rs542208825 | 0.000024 | 348 | A-band | 100 | 0.46444 |
| 738 | 62 | F | HL | c.56315C>T | p.Thr18772Ile | rs370118111 | 0.000008 | 289 | A-band | 100 | 0.57482 |
| 753 | 61 | M | NHB | c.9077A>T | p.Asn3026Ile | rs11900987 | 0.000454 | 38 | I-band | 100 | 0.56024 |
| 753 | 61 | M | NHB | c.82411G>A | p.Gly27471Ser | rs757130634 | |  | A-band | 100 | 0.31778 |
| 761 | 73 | M | NHB | c.98893G>C | p.Asp32965His | rs186405108 | 0.000213 | 353 | A-band | 100 | 0.70276 |
| 769 | 72 | M | NHB | c.72137C>T | p.Ala24046Val | rs146767076 | 0.000363 | 326 | A-band | 100 | 0.20419 |
| 769 | 72 | M | NHB | c.72782G>A | p.Arg24261Gln | rs142874389 | 0.000574 | 326 | A-band | 100 | 0.65522 |
| 769 | 72 | M | NHB | c.55951G>A | p.Glu18651Lys | |  |  | A-band | 100 | 0.6251 |
| 769 | 72 | M | NHB | c.44965A>G | p.Ile14989Val | rs755040094 | 0.000004 | 244 | I-band | 100 | 0.50496 |
| 769 | 72 | M | NHB | c.88973T>C | p.Ile29658Thr | rs750026544 | 0.000024 | 333 | A-band | 100 | 0.16972 |
| 781 | 61 | M | NHB | c.106439A>G | p.His35480Arg | rs766337455 | |  | M-band | 100 | 0.25558 |
| 820 | 59 | F | NHB | c.69383C>A | p.Ser23128Tyr | rs72646882 | 0.000576 | 324 | A-band | 100 | 0.76554 |
| 821 | 75 | F | NHB | c.47770T>A | p.Leu15924Met | |  |  | A-band | 100 | 0.83636 |
| 823 | 27 | M | HL | c.64997C>T | p.Ala21666Val | rs1396380194 | 0.000004 | 311 | A-band | 100 | 0.5065 |
| 823 | 27 | M | HL | c.106349C>G | p.Thr35450Ser | rs371022420 | 0.000045 | 358 | A-band | 100 | 0.30369 |
| 823 | 27 | M | HL | c.97760G>C | p.Arg32587Pro | rs55704830 | 0.000393 | 350 | A-band | 100 | 0.58633 |
| 823 | 27 | M | HL | c.57165A>T | p.Glu19055Asp | rs1263660973 | 0.000004 | 293 | A-band | 100 | 0.58507 |
| 823 | 27 | M | HL | c.73168A>G | p.Thr24390Ala | rs182491843 | 0.000481 | 326 | A-band | 100 | 0.08068 |
| 823 | 27 | M | HL | c.76141G>A | p.Ala25381Thr | rs763636099 | 0.000008 | 326 | A-band | 100 | 0.84345 |
| 823 | 27 | M | HL | c.106827T>G | p.Ile35609Met | rs727504540 | 0.000337 | 360 | M-band | 100 | 0.53062 |
| 825 | 52 | F | HL | c.88394C>T | p.Ser29465Phe | rs146181116 | 0.002928 | 331 | A-band | 100 | 0.65325 |
| 856 | 61 | F | NHB | c.79612A>G | p.Thr26538Ala | rs150682764 | 0.000322 | 326 | A-band | 100 | 0.70029 |
| 863 | 67 | M | HL | c.84309C>G | p.His28103Gln | rs749278779 | 0.000004 | 326 | A-band | 100 | 0.35528 |
| 868 | 62 | M | NHB | c.44077C>T | p.Arg14693Cys | rs200445568 | 0.000169 | 238 | I-band | 100 | 0.68844 |
| 868 | 62 | M | NHB | c.6927T>A | p.Asn2309Lys | rs147580120 | 0.000024 | 30 | I-band | 100 | 0.2335 |
| 868 | 62 | M | NHB | c.81539T>C | p.Ile27180Thr | rs182126530 | 0.000669 | 326 | A-band | 100 | 0.64318 |
| 885 | 66 | M | NHB | c.47737C>T | p.Leu15913Phe | rs138576504 | 0.000399 | 254 | A-band | 100 | 0.60456 |
| 885 | 66 | M | NHB | c.55547T>C | p.Ile18516Thr | rs146608896 | 0.000467 | 287 | A-band | 100 | 0.78662 |
| 895 | 64 | M | NHB | c.87611C>G | p.Thr29204Arg | rs72648228 | 0.000157 | 328 | A-band | 100 | 0.29647 |
| 895 | 64 | M | NHB | c.2764C>T | p.Arg922Cys | rs72647862 | 0.000331 | 16 | near Z-disk | 99 | 0.54641 |
| 895 | 64 | M | NHB | c.86393G>A | p.Arg28798Lys | rs781458689 | 0.000008 | 326 | A-band | 100 | 0.62838 |
| 895 | 64 | M | NHB | c.62432A>G | p.Asp20811Gly | rs72646849 | 0.000165 | 304 | A-band | 100 | 0.76487 |
| 896 | 72 | F | HL | c.57145G>A | p.Val19049Ile | rs750251277 | 0.000080 | 293 | A-band | 100 | 0.56694 |
| 896 | 72 | F | HL | c.92444G>A | p.Cys30815Tyr | rs1185347998 | 0.000004 | 339 | A-band | 100 | 0.59375 |
| 903 | 82 | M | HL | c.8509A>G | p.Ser2837Gly | rs202024134 | 0.000004 | 36 | I-band | 100 | 0.43662 |
| 903 | 82 | M | HL | c.86759C>G | p.Ser28920Cys | rs1396089552 | 0.000004 | 326 | A-band | 100 | 0.36365 |
| 903 | 82 | M | HL | c.92176C>T | p.Pro30726Ser | rs72648247 | 0.002682 | 339 | A-band | 100 | 0.79583 |
| 910 | 79 | M | NHB | c.101245G>A | p.Val33749Met | rs201554140 | 0.000538 | 358 | A-band | 100 | 0.72631 |
| 910 | 79 | M | NHB | c.4199G>C | p.Ser1400Thr | rs138506461 | 0.000518 | 24 | near Z-disk | 100 | 0.12618 |
| 910 | 79 | M | NHB | c.105127C>T | p.Arg35043Cys | rs200378865 | 0.000462 | 358 | A-band | 100 | 0.79765 |
| 932 | 48 | F | NHB | c.46040T>G | p.Val15347Gly | rs375367475 | 0.000044 | 248 | I-band | 100 | 0.77783 |
| 932 | 48 | F | NHB | c.72782G>A | p.Arg24261Gln | rs142874389 | 0.000574 | 326 | A-band | 100 | 0.65522 |
| 932 | 48 | F | NHB | c.72137C>T | p.Ala24046Val | rs146767076 | 0.000363 | 326 | A-band | 100 | 0.20419 |
| 938 | 67 | F | NHB | c.97106C>T | p.Thr32369Ile | rs559194338 | 0.000004 | 348 | A-band | 100 | 0.62729 |
| 943 | 78 | F | NHB | c.50390G>A | p.Arg16797His | rs200835354 | 0.000093 | 268 | A-band | 100 | 0.71084 |
| 956 | 66 | F | HL | c.94851T>A | p.Asp31617Glu | rs72648256 | 0.002881 | 342 | A-band | 100 | 0.6823 |
| 956 | 66 | F | HL | c.14870C>G | p.Thr4957Ser | rs72648925 | 0.002917 | 50 | I-band | 100 | 0.28911 |
| 969 | 68 | M | NHB | c.101936C>G | p.Pro33979Arg | rs200238877 | 0.000213 | 358 | A-band | 100 | 0.73517 |
| 969 | 68 | M | NHB | c.85691A>T | p.Lys28564Ile | rs199859344 | 0.000443 | 326 | A-band | 100 | 0.69443 |
| 969 | 68 | M | NHB | c.57683G>A | p.Arg19228His | rs114711705 | 0.000488 | 295 | A-band | 100 | 0.30369 |
| 970 | 65 | F | NHB | c.61138C>A | p.Leu20380Met | rs201167216 | 0.000271 | 304 | A-band | 100 | 0.41658 |
| 970 | 65 | F | NHB | c.86911G>A | p.Gly28971Arg | rs368921501 | 0.000040 | 327 | A-band | 100 | 0.79217 |
| 970 | 65 | F | NHB | c.66692G>A | p.Arg22231His | rs200971254 | 0.000343 | 316 | A-band | 100 | 0.76881 |
| 1016 | 65 | F | HL | c.69130C>T | p.Pro23044Ser | rs55980498 | 0.003619 | 324 | A-band | 100 | 0.83802 |

**Supplementary Table 1: List of *TTN* missense variants.** Age represents patient’s age at AF diagnosis in years. M=male, F=female, HL = Hispanic/Latinx, NHB = non-Hispanic Black. Variants with a blank value in the dbSNP or gnomAD columns represent variants not present in those respective databases.
