# Supplementary material for: A titin missense variant drives atrial electrical remodeling and is associated with atrial fibrillation
Source: eLife. 2026 Jan 22;14:RP104719. doi: 10.7554/eLife.104719 (PMC12826672; doi:10.7554/eLife.104719)
Supplement: Supplementary file 2. — *Data are missing for the following variables: eGFR (1), electrocardiogram within 3 months of AF diagnosis (11), LVEDD (19), left atrial size (6), left atrial diameter (21). Left ventricular dilatation is defined as left ventricular end diastolic diameter greater than 2 standard deviations above the normal sex-specific mean value. Variants with a REVEL score ≥0.7 were defined as predicted deleterious. Continuous data are represented as mean (standard deviation) and categorical data are represented as count (%) [file elife-104719-supp2.docx]

|  | **Predicted Deleterious *TTN* Missense Absent**  (N=88) | **Predicted Deleterious *TTN* Missense Present**  (N=43) | **Total**  (N=131) | **P-value** |
| --- | --- | --- | --- | --- |
| **Age at AF diagnosis (years)** | 63.7 (14.5) | 63.0 (12.5) | 63.5 (13.8) | 0.803 |
| **Male sex** | 47 (53.4%) | 23 (53.5%) | 70 (53.4%) | 1.000 |
| **Race/ethnicity** |  |  |  | 0.026 |
| Non-Hispanic Black | 57 (64.8%) | 36 (83.7%) | 93 (71.0%) |  |
| Hispanic/Latinx | 31 (35.2%) | 7 (16.3%) | 38 (29.0%) |  |
| **BMI (kg/m^2^)** | 33.8 (8.9) | 34.9 (11.1) | 34.1 (9.7) | 0.540 |
| **Diabetes** | 35 (39.8%) | 15 (34.9%) | 50 (38.2%) | 0.702 |
| **Hypertension** | 77 (87.5%) | 36 (83.7%) | 113 (86.3%) | 0.594 |
| **Coronary artery disease** | 19 (21.6%) | 13 (30.2%) | 32 (24.4%) | 0.288 |
| **History of stroke/transient ischemic attack** | 17 (19.3%) | 9 (20.9%) | 26 (19.8%) | 0.819 |
| **Congestive heart failure** | 32 (36.4%) | 22 (51.2%) | 54 (41.2%) | 0.131 |
| **Nonischemic dilated cardiomyopathy** | 6 (7.1%) | 6 (15.4%) | 12 (9.7%) | 0.191 |
| **Estimated glomerular filtration rate** (mg/dL) | 69.1 (24.6) | 68.9 (24.8) | 69.1 (24.6) | 0.965 |
| **Ventricular rate** | 90.9 (27.4) | 103.7 (31.4) | 95.3 (29.4) | 0.022 |
| **QRS interval (ms)** | 97.3 (24.3) | 102.7 (30.0) | 99.2 (26.3) | 0.292 |
| **QTc interval (ms)** | 453.9 (38.7) | 470.6 (44.0) | 459.6 (41.2) | 0.035 |
| **Left ventricular ejection fraction (%)** |  |  |  | 0.144 |
| Normal (>/=50%) | 55 (62.5%) | 22 (51.2%) | 77 (58.8%) |  |
| Mildly decreased (40-49%) | 11 (12.5%) | 3 (7.0%) | 14 (10.7%) |  |
| Moderately decreased (30-39%) | 7 (8.0%) | 6 (14.0%) | 13 (9.9%) |  |
| Severely decreased (20-29%) | 8 (9.1%) | 8 (18.6%) | 16 (12.2%) |  |
| Very severely decreased (< 20%) | 7 (8.0%) | 4 (9.3%) | 11 (8.4%) |  |
| **Left ventricular end diastolic diameter (mm)** | 45.6 (9.2) | 49.8 (8.0) | 46.9 (9.0) | 0.021 |
| **Left ventricular dilatation** | 9 (11.8%) | 8 (22.2%) | 17 (15.2%) | 0.168 |
| **Left atrial size** |  |  |  | 0.728 |
| Normal | 26 (31.3%) | 12 (28.6%) | 38 (30.4%) |  |
| Mildly dilated | 22 (26.5%) | 16 (38.1%) | 38 (30.4%) |  |
| Moderately dilated | 22 (26.5%) | 8 (19.0%) | 30 (24.0%) |  |
| Severely dilated | 13 (15.7%) | 6 (14.3%) | 19 (15.2%) |  |
| **Left atrial diameter (mm)** | 39.8 (7.5) | 41.5 (8.4) | 40.4 (7.8) | 0.293 |

**Supplementary Table 2:** **Clinical characteristics of ethnic minority subjects with AF stratified by presence of predicted deleterious rare missense *TTN* variants.** *Data are missing for the following variables: eGFR (1), electrocardiogram within 3 months of AF diagnosis (11), LVEDD (19), left atrial size (6), left atrial diameter (21). Left ventricular dilatation is defined as left ventricular end diastolic diameter greater than 2 standard deviations above the normal sex-specific mean value. Variants with a REVEL score ≥ 0.7 were defined as predicted deleterious. Continuous data are represented as mean (standard deviation) and categorical data are represented as count (%).
