# Supplementary material for: A titin missense variant drives atrial electrical remodeling and is associated with atrial fibrillation
Source: eLife. 2026 Jan 22;14:RP104719. doi: 10.7554/eLife.104719 (PMC12826672; doi:10.7554/eLife.104719)
Supplement: Supplementary file 3. — Nonischemic dilated cardiomyopathy was defined by left ventricular ejection fraction <50% and left ventricular end diastolic diameter (LVEDD) greater than 2 standard deviations above the sex-specific mean, as well as coronary angiogram confirming the absence of obstructive coronary artery disease. [file elife-104719-supp3.docx]

| **Subject ID** | **Age** | **Sex** | **Race-Ethnicity** | **Nucleotide** | **Amino Acid Change** | **Exon** | **Band** | **Percent Spliced**  **In (PSI)** | **REVEL**  **Score** | **LVEDD (mm)** | **LVEF (%)** |
| --- | --- | --- | --- | --- | --- | --- | --- | --- | --- | --- | --- |
| 39 | 56 | F | NHB | c.70250T>C | p.Ile23417Thr | 326 | A-band | 100 | 0.66297 | 57.9 | Mildly decreased (40-49%) |
| 65 | 43 | F | NHB | c.101665G>A | p.Val33889Ile | 358 | A-band | 100 | 0.17295 | 53.2 | Mildly decreased (40-49%) |
| 65 | 43 | F | NHB | c.6959G>A | p.Arg2320His | 30 | I-band | 100 | 0.82403 | 53.2 | Mildly decreased (40-49%) |
| 160 | 69 | F | NHB | c.91937A>G | p.Asn30646Ser | 338 | A-band | 100 | 0.71558 | 56.9 | Severely decreased (20-29%) |
| 160 | 69 | F | NHB | c.46693G>T | p.Ala15565Ser | 250 | I-band | 100 | 0.51417 | 56.9 | Severely decreased (20-29%) |
| 243 | 57 | M | NHB | c.44525C>T | p.Thr14842Ile | 241 | I-band | 100 | 0.75347 | 59.0 | Very severely decreased (< 20%) |
| 243 | 57 | M | NHB | c.81502C>T | p.Arg27168Cys | 326 | A-band | 100 | 0.63269 | 59.0 | Very severely decreased (< 20%) |
| 457 | 42 | F | NHB | c.69883G>A | p.Ala23295Thr | 326 | A-band | 100 | 0.21907 | 60.3 | Moderately decreased (30-39%) |
| 542 | 46 | M | NHB | c.101245G>A | p.Val33749Met | 358 | A-band | 100 | 0.72631 | 66.5 | Very severely decreased (< 20%) |
| 542 | 46 | M | NHB | c.4199G>C | p.Ser1400Thr | 24 | near Z-disk | 100 | 0.12618 | 66.5 | Very severely decreased (< 20%) |
| 542 | 46 | M | NHB | c.105127C>T | p.Arg35043Cys | 358 | A-band | 100 | 0.79765 | 66.5 | Very severely decreased (< 20%) |
| 823 | 27 | M | HL | c.64997C>T | p.Ala21666Val | 311 | A-band | 100 | 0.5065 | 60.3 | Severely decreased (20-29%) |
| 823 | 27 | M | HL | c.106349C>G | p.Thr35450Ser | 358 | A-band | 100 | 0.30369 | 60.3 | Severely decreased (20-29%) |
| 823 | 27 | M | HL | c.97760G>C | p.Arg32587Pro | 350 | A-band | 100 | 0.58633 | 60.3 | Severely decreased (20-29%) |
| 823 | 27 | M | HL | c.57165A>T | p.Glu19055Asp | 293 | A-band | 100 | 0.58507 | 60.3 | Severely decreased (20-29%) |
| 823 | 27 | M | HL | c.73168A>G | p.Thr24390Ala | 326 | A-band | 100 | 0.08068 | 60.3 | Severely decreased (20-29%) |
| 823 | 27 | M | HL | c.76141G>A | p.Ala25381Thr | 326 | A-band | 100 | 0.84345 | 60.3 | Severely decreased (20-29%) |
| 823 | 27 | M | HL | c.106827T>G | p.Ile35609Met | 360 | M-band | 100 | 0.53062 | 60.3 | Severely decreased (20-29%) |
| 885 | 66 | M | NHB | c.47737C>T | p.Leu15913Phe | 254 | A-band | 100 | 0.60456 | 66.2 | Moderately decreased (30-39%) |
| 885 | 66 | M | NHB | c.55547T>C | p.Ile18516Thr | 287 | A-band | 100 | 0.78662 | 66.2 | Moderately decreased (30-39%) |

**Supplementary Table 3: *TTN* missense variants in subjects meeting criteria for nonischemic dilated cardiomyopathy**. Nonischemic dilated cardiomyopathy was defined by left ventricular ejection fraction <50% and left ventricular end diastolic diameter (LVEDD) greater than 2 standard deviations above the sex-specific mean, as well as coronary angiogram confirming the absence of obstructive coronary artery disease.
