# Supplementary material for: A titin missense variant drives atrial electrical remodeling and is associated with atrial fibrillation
Source: eLife. 2026 Jan 22;14:RP104719. doi: 10.7554/eLife.104719 (PMC12826672; doi:10.7554/eLife.104719)
Supplement: Supplementary file 4. — A partially adjusted multivariable model contained covariates of age and sex, and the fully adjusted model additionally accounted for race-ethnicity and ejection fraction <50% closest to AF diagnosis. [file elife-104719-supp4.docx]

|  | Unadjusted | | | Partially Adjusted | | | Fully Adjusted | | |
| --- | --- | --- | --- | --- | --- | --- | --- | --- | --- |
| **Characteristic** | **HR***^1^* | **95% CI***^1^* | **p-value** | **HR***^1^* | **95% CI***^1^* | **p-value** | **HR***^1^* | **95% CI***^1^* | **p-value** |
| *TTN* Missense Present | 1.81 | 1.04, 3.15 | 0.036 | 1.82 | 1.04, 3.17 | 0.035 | 1.80 | 1.03, 3.15 | 0.039 |
| Age (years) |  |  |  | 0.99 | 0.97, 1.01 | 0.366 | 0.99 | 0.97, 1.01 | 0.430 |
| Male sex (vs. female) |  |  |  | 0.74 | 0.43, 1.27 | 0.268 | 0.69 | 0.38, 1.24 | 0.218 |
| Race-ethnicity |  |  |  |  |  |  |  |  |  |
| Non-Hispanic Black |  |  |  |  |  |  | — | — |  |
| Hispanic/Latinx |  |  |  |  |  |  | 1.14 | 0.60, 2.19 | 0.683 |
| Baseline ejection fraction <50% |  |  |  |  |  |  | 1.38 | 0.78, 2.44 | 0.272 |
| *^1^*HR = Hazard Ratio, CI = Confidence Interval | | | | | | | | | |

**Supplementary Table 4: Parameter estimates for univariable and multivariable Cox proportional hazard models of atrial fibrillation and heart failure-related hospitalizations.** A partially adjusted multivariable model contained covariates of age and sex, and the fully adjusted model additionally accounted for race-ethnicity and ejection fraction <50% closest to AF diagnosis.
