# Supplementary material for: A titin missense variant drives atrial electrical remodeling and is associated with atrial fibrillation
Source: eLife. 2026 Jan 22;14:RP104719. doi: 10.7554/eLife.104719 (PMC12826672; doi:10.7554/eLife.104719)
Supplement: Supplementary file 5. — REVEL score of ≥0.70 indicates potentially deleterious effect. A partially adjusted multivariable model contained covariates of age and sex, and the fully adjusted model additionally accounted for race-ethnicity and ejection fraction <50% closest to AF diagnosis. [file elife-104719-supp5.docx]

|  | Unadjusted | | | Partially Adjusted | | | Fully Adjusted | | |
| --- | --- | --- | --- | --- | --- | --- | --- | --- | --- |
| **Characteristic** | **HR***^1^* | **95% CI***^1^* | **p-value** | **HR***^1^* | **95% CI***^1^* | **p-value** | **HR***^1^* | **95% CI***^1^* | **p-value** |
| *TTN* Missense |  |  |  |  |  |  |  |  |  |
| None | — | — |  | — | — |  | — | — |  |
| REVEL <0.70 | 1.60 | 0.78, 3.28 | 0.198 | 1.61 | 0.79, 3.26 | 0.188 | 1.60 | 0.80, 3.21 | 0.182 |
| REVEL ≥0.70 | 1.92 | 1.04, 3.53 | 0.036 | 1.92 | 1.04, 3.56 | 0.038 | 1.91 | 1.04, 3.51 | 0.038 |
| Age (years) |  |  |  | 0.99 | 0.97, 1.01 | 0.363 | 0.99 | 0.97, 1.01 | 0.412 |
| Male sex (vs. female) |  |  |  | 0.73 | 0.42, 1.25 | 0.251 | 0.68 | 0.38, 1.23 | 0.204 |
| Race-ethnicity |  |  |  |  |  |  |  |  |  |
| Non-Hispanic Black |  |  |  |  |  |  | — | — |  |
| Hispanic/Latinx |  |  |  |  |  |  | 1.17 | 0.63, 2.16 | 0.619 |
| Baseline ejection fraction <50% |  |  |  |  |  |  | 1.35 | 0.77, 2.36 | 0.302 |
| *^1^*HR = Hazard Ratio, CI = Confidence Interval | | | | | | | | | |

**Supplementary Table 5: Cox proportional hazard models of hospitalizations related to TTN missense variants based on *in silico* prediction of impact.** REVEL score of ≥0.70 indicates potentially deleterious effect. A partially adjusted multivariable model contained covariates of age and sex, and the fully adjusted model additionally accounted for race-ethnicity and ejection fraction <50% closest to AF diagnosis.
