# Supplementary material for: A titin missense variant drives atrial electrical remodeling and is associated with atrial fibrillation
Source: eLife. 2026 Jan 22;14:RP104719. doi: 10.7554/eLife.104719 (PMC12826672; doi:10.7554/eLife.104719)
Supplement: Supplementary file 6. — A total of 12 subjects were excluded. A partially adjusted multivariable model contained covariates of age and sex, and the fully adjusted model additionally accounted for race-ethnicity and ejection fraction <50% closest to AF diagnosis. [file elife-104719-supp6.docx]

|  | Unadjusted | | | Partially Adjusted | | | Fully Adjusted | | |
| --- | --- | --- | --- | --- | --- | --- | --- | --- | --- |
| **Characteristic** | **HR***^1^* | **95% CI***^1^* | **p-value** | **HR***^1^* | **95% CI***^1^* | **p-value** | **HR***^1^* | **95% CI***^1^* | **p-value** |
| *TTN* Missense Present | 1.81 | 1.00, 3.29 | 0.051 | 1.84 | 1.01, 3.34 | 0.046 | 1.83 | 1.01, 3.32 | 0.046 |
| Age (years) |  |  |  | 0.99 | 0.97, 1.01 | 0.365 | 0.99 | 0.97, 1.01 | 0.360 |
| Male sex (vs. female) |  |  |  | 0.75 | 0.42, 1.35 | 0.344 | 0.70 | 0.37, 1.32 | 0.268 |
| Race-ethnicity |  |  |  |  |  |  |  |  |  |
| Non-Hispanic Black |  |  |  |  |  |  | — | — |  |
| Hispanic/Latinx |  |  |  |  |  |  | 1.17 | 0.59, 2.31 | 0.649 |
| Baseline ejection fraction <50% |  |  |  |  |  |  | 1.41 | 0.76, 2.61 | 0.280 |
| *^1^*HR = Hazard Ratio, CI = Confidence Interval | | | | | | | | | |

**Supplementary Table 6: Cox proportional hazard models of hospitalizations excluding cases with nonischemic dilated cardiomyopathy.** A total of 12 subjects were excluded. A partially adjusted multivariable model contained covariates of age and sex, and the fully adjusted model additionally accounted for race-ethnicity and ejection fraction <50% closest to AF diagnosis.
