# Supplementary material for: A titin missense variant drives atrial electrical remodeling and is associated with atrial fibrillation
Source: eLife. 2026 Jan 22;14:RP104719. doi: 10.7554/eLife.104719 (PMC12826672; doi:10.7554/eLife.104719)
Supplement: Supplementary file 7. [file elife-104719-supp7.docx]

| Nucleotide and Protein ID | NM_001267550.2(TTN):c.98267C>T ( NP_001254479.2:p.Thr32756Ile) |
| --- | --- |
| Allele ID | 173049 |
| Variant type | single nucleotide variant (missense) |
| Variant length | 1 bp |
| Cytogenetic location | 2q31.2 |
| Genomic location | 2: 178539798 (GRCh38) GRCh38 UCSC; 2: 179404525 (GRCh37) GRCh37 UCSC |
| Canonical SPDI | NC_000002.12:178539797:G:A |
| Source | https://www.ncbi.nlm.nih.gov/clinvar/variation/178164/ |

**Supplementary Table 7: *TTN-*T32756I variant information.**
